# A Curcumin‐Based Polyherbal Slow‐Dissolving Buccal Lozenge (CBFP‐ODS) and Changes in Gingival Inflammation, Plaque Accumulation, Bleeding on Probing and Oral Malodour: A 12‐Week Prospective Single‐Arm Interventional Study in Young Adults

**DOI:** 10.1155/ijod/2236774

**Published:** 2026-11-10

**Authors:** Vaibhav Kumar, L. N. Dedova, Nupur Sah, Nirupam K. Doctor, Manasi Kamble

**Affiliations:** ^1^ Department of Public Health Dentistry Dr. G. D. Pol Foundation Y.M.T. Dental College and Hospital Navi Mumbai Maharashtra India; ^2^ Department of Public Health Dentistry Manipal College of Dental Sciences, Mangalore Manipal Academy of Higher Education Manipal India manipal.edu; ^3^ Department of Periodontology Belarusian State Medical University Minsk Belarus bsmu.by; ^4^ Department of Periodontology Dr. G. D. Pol Foundation Y.M.T. Dental College and Hospital Navi Mumbai Maharashtra India; ^5^ Doctor’s Dental Clinic Mumbai India

**Keywords:** buccal lozenge, curcumin, gingivitis, oral hygiene, phytotherapy, preventive dentistry, single-arm study

## Abstract

**Background:**

Plaque‐induced gingivitis is the most prevalent reversible inflammatory condition of the gingival tissues and remains under‐managed at the population level. Chlorhexidine, the most extensively studied chemical adjunct, is limited in sustained daily use by staining, taste alteration and mucosal effects, which motivates interest in botanically based oral wellness adjuncts intended for everyday use. This study was an uncontrolled, exploratory, hypothesis‐generating evaluation of one such adjunct.

**Methods:**

We evaluated a curcumin‐based functional phytotherapeutic oral delivery system (CBFP‐ODS), a sugar‐free, slow‐dissolving buccal lozenge containing six standardised botanicals (*Curcuma longa*, *Ocimum sanctum*, *Zingiber officinale*, *Cinnamomum verum*, *Mentha piperita* and *Piper longum*), in adults aged 18–35 years with mild‐to‐moderate gingivitis. In a prospective, single‐arm, open‐label, pre‐ and post‐interventional study (120 enrolled), participants used one lozenge twice daily for 12 weeks while continuing routine mechanical plaque control. Outcomes at five timepoints (baseline, 1 week, 21 days, 45 days, 12 weeks) included the modified gingival index (MGI; primary outcome), the Silness and Löe Plaque Index (PI), the Ainamo and Bay Bleeding on Probing (BOP) index, an examiner‐rated organoleptic score, an adapted breath‐related self‐perception (BRSP) scale and a WHO‐adapted oral hygiene and behaviour composite. The pre‐specified primary analysis was a completer analysis of the 115 participants with complete follow‐up; a mixed‐effects model on all 120 enrolled participants was the sensitivity analysis.

**Results:**

In the primary completer analysis (*n* = 115), MGI decreased from 1.78 (SD 0.41) to 0.96 (SD 0.36) (mean change 0.82, 95% CI: 0.73–0.91; *t* (114) = 18.9; *p* < 0.001; Cohen’s dz ~1.76). Plaque, BOP, oral malodour and self‐perceived breath showed within‐subject reductions of ~43%–53%, and the behavioural composite increased by ~31% (all *p* < 0.001). The mixed‐effects sensitivity analysis on all 120 enrolled participants gave a concordant estimated reduction of 0.81 MGI units (95% CI: 0.73–0.89). No product‐related adverse events were recorded.

**Conclusions:**

In this single‐arm study, twice‐daily use of CBFP‐ODS was associated with within‐subject improvements in gingival, plaque, bleeding and breath‐related outcomes over 12 weeks. Because the design was uncontrolled and open‐label, these observations cannot separate the effect of the intervention from improved oral hygiene behaviour, participation effects, regression to the mean or natural clinical variation and are hypothesis‐generating. They do not demonstrate efficacy or verify mucosal absorption. Randomised, placebo‐controlled and active‐controlled trials are required before any statement about efficacy can be made.

**Trial Registration:** Clinical Trials Registry, India: (CTRI/2025/06/088964; prospectively registered)

## 1. Introduction

Plaque‐induced gingivitis is a reversible inflammatory condition of the gingival tissues initiated by the accumulation of microbial dental plaque at the gingival margin. It is recognised as a distinct case definition in the 2017 World Workshop classification of periodontal and peri‐implant diseases and conditions and remains one of the most prevalent conditions in human health, affecting a substantial majority of adolescents and young adults in epidemiological surveys [1–3]. Although clinically reversible, untreated gingivitis is the most consistent clinical antecedent of periodontitis and has been linked to systemic low‐grade inflammation [3, 4].

Day‐to‐day management of gingivitis remains anchored in mechanical plaque control, supported when necessary by chemical mouthrinses. Chlorhexidine gluconate is the most extensively studied chemical adjunct and produces consistent reductions in plaque accumulation and gingival inflammation in randomised trials and systematic reviews [5, 6]. Its long‐term utility is nonetheless constrained by a well‐documented profile of side effects, including extrinsic tooth and restoration staining, transient taste alteration and mucosal irritation, that limit compliance and in practice restrict it to short therapeutic courses [5–7]. These considerations motivate interest in botanically based oral wellness formats intended for regular use. The present study did not assess comparative tolerability, and no claim of a tolerability or safety advantage over chlorhexidine is made or implied; chlorhexidine is referred to here only as the reference chemical adjunct that frames the rationale for evaluating daily‐use alternatives, and was not included as a comparator.

Interest in phytochemicals for oral health has expanded substantially over the past two decades. Curcumin, the principal bioactive constituent of *Curcuma longa* (turmeric), has been studied for its modulation of inflammatory signalling, including inhibition of nuclear factor‐κB (NF‐κB), cyclooxygenase‐2 (COX‐2) and pro‐inflammatory cytokines such as IL‐1β and TNF‐α, and for its antioxidant and antimicrobial activity in periodontal contexts [8–10]. These mechanisms are cited here as biological plausibility drawn from prior laboratory and clinical work, and not as mechanisms demonstrated in the present study, which measured clinical and patient‐reported endpoints only. Clinical evaluations of curcumin in gingival applications have shown anti‐inflammatory and anti‐plaque effects, with results varying by formulation and duration of mucosal contact [11, 12].

Two translational gaps persist. First, most curcumin preparations evaluated for oral indications have been formulated as gels or short‐contact rinses. Rinse‐based delivery is intrinsically limited by salivary dilution, swallowing and rapid clearance [13]. Curcumin is also poorly bioavailable in standard preparations because of rapid hepatic and intestinal glucuronidation; co‐administration with piperine has been shown, in pharmacokinetic studies, to enhance its absorption [14, 15]. A slow‐dissolving buccal format is intended to prolong the local contact time between the phytochemical matrix and the gingival margin relative to a rinse. It is important to state that prolongation of local contact is a design intention only: the present study did not measure mucosal absorption, tissue concentrations or systemic pharmacokinetics and therefore does not demonstrate enhanced tissue exposure or bioavailability. Second, oral phytotherapy has rarely been studied as a single, polyherbal, daily‐use format with a defined delivery vehicle and behavioural reinforcement, which is the form factor most relevant to community oral health practice.

The curcumin‐based functional phytotherapeutic oral delivery system (CBFP‐ODS) evaluated here was designed to address both gaps. It combines six standardised botanicals (*Curcuma longa*, *Ocimum sanctum*, *Zingiber officinale*, *Cinnamomum verum*, *Mentha piperita* and *Piper longum*) selected for complementary anti‐inflammatory, antimicrobial, immunomodulatory and bioavailability‐enhancing properties reported in prior literature [9, 10, 14, 16–18], and is delivered as a sugar‐free, slow‐dissolving lozenge intended for prolonged local contact. The intention is not to replace mechanical plaque control or short‐course chemical adjuncts where indicated, but to provide a daily‐use wellness adjunct that may support gingival health in everyday life; this potential role is presented as a hypothesis for future controlled testing rather than an established use.

Against this backdrop, the present study evaluated the clinical changes observed during the twice‐daily use of CBFP‐ODS for 12 weeks in adults with mild‐to‐moderate gingivitis. The study hypothesis, stated a priori in the registered protocol, was that within‐subject gingival, plaque, bleeding and breath‐related indices would show measurable improvement across the 12‐week window. A single‐arm design was chosen deliberately as a first, hypothesis‐generating step to establish feasibility, adherence, tolerability and the plausible direction and magnitude of within‐subject change for a newly formulated product, before committing the resources required for a randomised, placebo‐controlled trial. This design choice, and the inferential limits it imposes, are acknowledged throughout. The study was prospectively registered and is reported in line with reporting principles applicable to non‐randomised single‐arm interventional studies [19].

## 2. Materials and Methods

### 2.1. Study Design and Reporting

This was a prospective, non‐randomised, single‐arm, open‐label interventional study employing a pre and post (within‐participant) repeated‐measures design. All enrolled participants received the investigational intervention, and each participant served as their own comparison, with outcomes assessed at baseline (T0) and at four scheduled follow‐up visits (T1: 1 week, T2: 21 days, T3: 45 days and T4: 12 weeks). A within‐participant comparison is not equivalent to a concurrent control group and does not permit causal attribution. The study was conducted in line with the Declaration of Helsinki (2013 revision) and reported in accordance with reporting principles for non‐randomised interventional studies, drawing on applicable CONSORT 2010 items [19]. The flow of participants is summarised in Figure 1.

**Figure 1:**
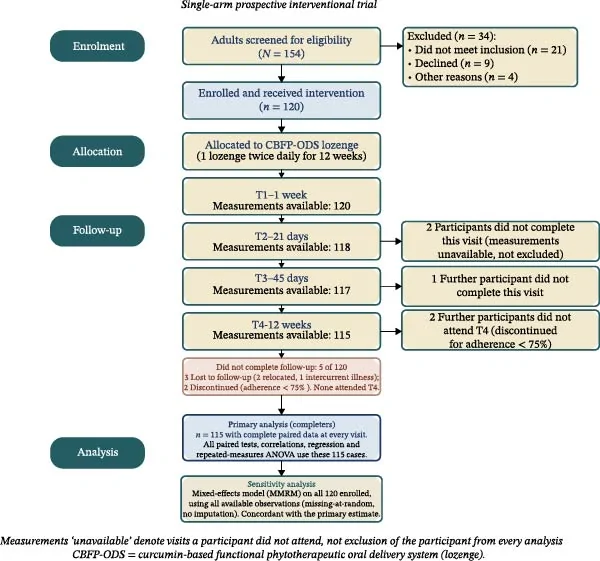
Flow of participants. Of 154 screened, 120 were enrolled and 115 completed all five visits and formed the primary (completer) analysis population. Intermediate boxes report the number of observed measurements available at each visit; a participant not attending a visit produces an unavailable measurement and is not the same as excluding that participant from every analysis. The primary analysis (paired tests, correlations, regression, repeated‐measures ANOVA) used the 115 completers; a mixed‐effects model on all 120 enrolled participants, under a missing‐at‐random assumption and without imputation, was the sensitivity analysis and was concordant.

### 2.2. Ethical Approval and Trial Registration

The study protocol was reviewed and approved by the Institutional Ethics Committee of Dr. G. D. Pol Foundation Y.M.T. Dental College and Hospital, Kharghar, Navi Mumbai (Indian Council of Medical Research and Department of Health Research registration EC/NEW/INST/2023/3747), prior to participant enrolment. The approval was issued under reference number YMTDC/IEC/OUT/2025/329‐P, dated 12 June 2025. The trial was prospectively registered with the Clinical Trials Registry, India, before the first participant visit. Written informed consent was obtained from all participants, in their preferred or native language, prior to any study procedure. Confidentiality was maintained through coded participant identifiers and restricted dataset access. Participants were free to withdraw at any time without giving a reason and without any impact on their access to standard oral health care.

### 2.3. Setting and Study Period

Screening, enrolment, intervention delivery and clinical outcome assessment were conducted in a standardised dental clinical setting in Navi Mumbai and Mumbai, India. Participant recruitment and baseline enrolment took place between 7 July 2025 and 22 August 2025. Each enrolled participant was then followed for 12 weeks from their own baseline, so that end‐of‐study (T4) assessments extended to mid‐November 2025; the final participant completed the 12‐week visit on 14 November 2025. Recruitment dates are thus distinct from the date of the last follow‐up visit, and the overall study window accommodates a full 12 weeks of observation for the last enrolled participant.

### 2.4. Participants

The study population comprised adults aged 18–35 years of any gender with clinically confirmed mild‐to‐moderate gingivitis. The age band was selected for epidemiological comparability with internationally used WHO oral health index age groupings [20]. Participants were recruited consecutively from the catchment area until the predetermined sample size was reached. The eligibility criteria are listed below.

#### 2.4.1. Inclusion Criteria

• Adult aged 18–35 years.
• Mild‐to‐moderate gingivitis, operationalised as a modified gingival index (MGI) score between 1.0 and 2.5 [21].
• Presence of at least 20 natural teeth, excluding third molars.
• Willing and able to maintain routine oral hygiene practices for the duration of the study.
• Provided written informed consent in their preferred or native language.
• Agreed to abstain from medicated mouthrinses, additional herbal oral products and oral health‐related supplements during the follow‐up period.

#### 2.4.2. Exclusion Criteria

• Clinical periodontitis (clinical attachment loss of 3 mm or more at two or more non‐adjacent teeth, or radiographic evidence of alveolar bone loss).
• Use of systemic antibiotics, anti‐inflammatory medication or corticosteroids within the preceding 3 months.
• Current smoking or smokeless tobacco use.
• Pregnancy or lactation.
• Known allergy or hypersensitivity to any botanical constituent of the investigational product.
• Systemic disease or therapy known to influence periodontal health (for example, poorly controlled diabetes mellitus with HbA1c above 8%, autoimmune disease or immunosuppressive therapy).
• Use of any herbal, homeopathic or ayurvedic oral health product within 30 days of enrolment.
• Participation in any other interventional clinical study within the prior 3 months.

### 2.5. Intervention (CBFP‐ODS)

The investigational product was a standardised polyherbal lozenge (Dennkur) formulated as a sugar‐free, slow‐dissolving solid intended to release its actives in the buccal cavity over several minutes, prolonging local contact between the phytochemical matrix and the gingival margin relative to a rinse. Prolongation of local contact is a formulation objective; the study did not measure mucosal absorption or tissue exposure.

#### 2.5.1. Composition, Standardisation and Quality Control

Each lozenge contained six standardised botanical extracts at fixed quantities: *Curcuma longa* (turmeric) rhizome extract 150 mg, standardised to not less than 95% total curcuminoids (~142.5 mg curcuminoids per lozenge); *Ocimum sanctum* (holy basil) leaf extract 25 mg, standardised to not less than 2.5% ursolic acid; *Zingiber officinale* (ginger) rhizome extract 50 mg, standardised to not less than 5% gingerols; *Cinnamomum verum* (cinnamon) bark extract 10 mg, standardised to not less than 8% total polyphenols; *Mentha piperita* (peppermint) leaf extract 10 mg, standardised to not less than 30% total menthol; and *Piper longum* (long pepper) fruit extract 5 mg, standardised to not less than 95% piperine (~4.75 mg piperine per lozenge), included specifically as a bioavailability enhancer. The total standardised botanical load was ~250 mg per lozenge, carried in a sugar‐free, slow‐dissolving excipient base (isomalt and sorbitol bulk sweeteners, acacia and hydroxypropyl methylcellulose as matrix formers, magnesium stearate as lubricant, and natural peppermint flavour). Marker‐compound content (total curcuminoids and piperine) was verified for each production batch by high‐performance liquid chromatography (HPLC) against reference standards, and identity of the botanical extracts was confirmed by thin‐layer chromatography; each batch was additionally checked for uniformity of weight, hardness, appearance and disintegration time. The botanicals were standardised in line with the Ayurvedic Pharmacopoeia of India. The full batch quality‐control specifications, and the manufacturer and manufacturing‐licence details are provided in the Supporting Information (Table S1); the manufacturer is also identified in the Title Page file.

A commercial oral‐care product later derived from this delivery platform uses a reformulated botanical composition; the formulation described above is the one prepared and used in the present trial, and readers should not assume identity between the trial formulation and any later marketed product.

#### 2.5.2. Provenance, Supply and Investigator Relationship

This was an investigator‐initiated study. The trial lozenges were prepared to the fixed specification above by a licensed contract manufacturer under an Ayurvedic manufacturing licence and were supplied for research use; the manufacturer and licence identifiers are given in the Title Page file. No author, department or institution holds any financial interest, commercial relationship, advisory arrangement, product‐supply agreement, or intellectual‐property or formulation‐rights stake connected to the product, and no commercial entity had any role in the conception, design, conduct, data collection, analysis, interpretation, writing or the decision to submit this work. The costs of preparing and supplying the trial lozenges were met by the investigators as part of this investigator‐initiated study, and no external funding of any kind was received. The investigators had full and independent control over every stage. These disclosures are restated in the Conflicts of Interest and Funding statements and are consistent with them.

#### 2.5.3. Administration and Standardisation of Concomitant Oral Care

Participants were instructed to administer one lozenge twice daily, after breakfast and after the evening meal, for 12 weeks and to allow the lozenge to dissolve slowly without chewing. To limit confounding, all participants received identical structured oral hygiene instruction at baseline and were provided with a standard soft‐bristled manual toothbrush and a standard 1450 ppm sodium fluoride dentifrice, with instruction to brush twice daily using a modified Bass technique. No professional scaling or prophylaxis was performed during the study period, and interdental cleaning advice was standardised to once‐daily interdental brush use for all participants. Participants abstained from medicated mouthrinses, other herbal oral‐care products and non‐study oral health supplements during follow‐up. Because standardised oral hygiene instruction and mechanical care were provided to every participant, any improvement observed cannot be attributed to the lozenge in isolation; this is addressed in Section 4.6.

### 2.6. Outcome Measures

#### 2.6.1. Primary Outcome

The primary outcome was the within‐subject change in gingival inflammation, assessed using the MGI of Lobene et al. [21] over 12 weeks, with emphasis on the baseline‐to‐12‐week contrast. MGI was scored at six sites per tooth (mesiobuccal, mid‐buccal, distobuccal, mesiolingual, mid‐lingual and distolingual) on all fully erupted teeth excluding third molars (full‐mouth assessment). Duplicate MGI scoring was performed at baseline on all participants to support the reliability estimates reported in Section 2.7; at follow‐up visits, a single full‐mouth assessment was recorded per participant.

#### 2.6.2. Secondary Outcomes and Measurement Procedures

Secondary outcomes were assessed full‐mouth by the same examiner at each visit. Two established clinical indices and one examiner‐rated malodour score were used, together with two patient‐reported instruments that were adapted for this study (described below); the clinical indices should therefore be understood as validated instruments and the two patient‐reported instruments as adapted instruments.

• Plaque accumulation: Silness and Löe Plaque Index (PI), scored at four surfaces per tooth (buccal, lingual, mesial and distal) [22].
• Gingival bleeding tendency: Ainamo and Bay Bleeding on Probing index (BOP), expressed as the percentage of bleeding sites per participant, recorded at six sites per tooth using a University of North Carolina (UNC‐15) periodontal probe at a standardised probing force of ~0.25 N, with bleeding read 20 s after probing.
• Oral malodour (examiner‐rated): an examiner‐rated organoleptic score (0–5) [23]. This is a subjective examiner rating and is not an instrumental measurement; volatile sulphur compounds were not measured instrumentally, which is acknowledged as a limitation. Assessment was standardised: participants abstained from eating, drinking (except water), smoking, chewing gum and using scented cosmetics for 2 h beforehand, from antibiotics for the preceding 3 weeks, and from the study lozenge and any oral hygiene procedure for at least 3 h before assessment, so that recent product use did not influence the score. Assessment was performed between 09:00 and 11:00 by a single calibrated examiner who scored the expired air at a fixed distance of 10 cm from the participant’s mouth.
• Self‐perceived breath (patient‐reported, adapted): a breath‐related self‐perception (BRSP) scale.
• Oral hygiene knowledge, practices and self‐perceived gingival health (patient‐reported, adapted): a WHO‐adapted Gingival Health and Oral Hygiene Questionnaire structured for pre‐ and post‐behavioural surveillance [24].

The BRSP scale and the WHO‐adapted composite were adapted or newly assembled for this study rather than used as previously validated instruments. Their complete items, response options, total scoring ranges, direction of scoring and the derivation of each composite score are provided in full in the Supporting Information (Appendix S1 for the BRSP scale and Appendix S2 for the WHO‐adapted composite). In brief, the BRSP scale comprises six self‐rated items on breath confidence and social impact, each scored 0–10 and averaged to a 0–10 score, with a higher score denoting worse self‐perceived breath. The WHO‐adapted composite comprises 12 knowledge, practice and self‐perception items summed to a 0–40 score, with a higher total denoting better oral hygiene and self‐perceived gingival health. Internal consistency was estimated at baseline (T0) and was acceptable, with Cronbach’s alpha of 0.82 for the BRSP scale and 0.79 for the WHO‐adapted composite. Because these instruments were adapted, findings based on them are interpreted with corresponding caution.

### 2.7. Examiner Training, Calibration and Reliability

All clinical indices were recorded by a single trained examiner using a custom‐coded clinical examination form. Prior to study commencement, the examiner was calibrated against a reference examiner on a separate calibration sample of 15 volunteers with mild‐to‐moderate gingivitis who were not enrolled in the study. Each calibration participant was assessed twice, at a 1 h interval, for all indices. Intra‐examiner reliability on the calibration sample was high: the weighted kappa was 0.86 for MGI and 0.84 for the PI, the percentage agreement for site‐level BOP was 91% (kappa 0.82), and the intraclass correlation coefficient for the organoleptic score was 0.88. A pre‐specified threshold of kappa above 0.80 was met before outcome recording commenced. Duplicate baseline scoring of enrolled participants (Section 2.6.1) gave concordant intra‐examiner reliability (MGI intraclass correlation, 0.90). Blinding of the examiner to timepoint was not feasible in a longitudinal within‐participant design, and single‐examiner assessment precludes inter‐examiner reliability estimation; both are acknowledged in Section 4.6.

### 2.8. Adherence Monitoring, Retention and Safety

Adherence was monitored using a daily lozenge diary completed by participants and weekly telephone follow‐up calls. Adherence was therefore self‐reported and diary‐based and was not biologically verified, which is stated as a limitation. Adherence was expressed as the proportion of prescribed doses recorded as taken. Safety was monitored continuously through diaries, telephone calls and clinic visits. Participants were asked to report any adverse event, whether or not thought related to the product, including mucosal irritation, hypersensitivity, gastrointestinal symptoms and any other unexpected symptom. Each event was documented with onset, duration, severity, action taken, outcome and investigator‐assessed relatedness. Relatedness was judged using standard temporal and dechallenge criteria: events with no plausible temporal link to lozenge use, an identifiable alternative cause and no association with the local site of application were classified as unrelated.

### 2.9. Sample Size Calculation

The sample size was determined a priori, before recruitment, and is documented in the registered protocol. The calculation used G ^∗^Power Version 3.1 [25] for the primary outcome (within‐subject change in MGI), employing the paired‐sample means procedure (difference between two dependent means). Because the paired test is governed by the standard deviation of the within‐subject difference score, the calculation was based on that quantity rather than on a raw within‐subject standard deviation. A difference‐score standard deviation of 0.52 MGI units and a minimal clinically relevant mean change of 0.15 MGI units were assumed. These values were taken from the within‐group pre‐to‐post change and its dispersion reported for the curcumin arms of prior controlled gingival trials [11, 12], from which the ratio of the change to its standard deviation was derived and applied conservatively; they were the protocol assumptions specified before recruitment and are not a post hoc justification. With a two‐sided alpha of 0.05 and a target power of 80%, the procedure returned a required completer sample of 97 participants (G ^∗^Power inputs: test family = *t*‐tests; statistical test = means, difference between two dependent means (matched pairs); type of power analysis = a priori; tails = two; effect size dz = 0.288; alpha = 0.05; power = 0.80; output: noncentrality parameter delta = 2.836, critical *t* = 1.985, df = 96, actual power = 0.802). To allow for ~20% attrition, a target of 120 participants was enrolled. The 115 participants who completed follow‐up exceeded the required completer sample and corresponded to a post hoc power of ~0.86 for the primary within‐subject contrast at the assumed effect size. The full G ^∗^Power protocol and output are provided in the Supporting Information (Table S2).

### 2.10. Data Management and Statistical Analysis

Study data were captured on structured case‐record forms and digitised after each visit, with range checks and verification of improbable values against the source records. Analyses used IBM SPSS Statistics Version 29.0; figures were generated in R Version 4.3.1. Continuous variables were summarised as mean (SD), or median (interquartile range) where appropriate, and categorical variables as frequencies and percentages. The normality of within‐subject change scores was assessed using the Shapiro and Wilk test with graphical inspection.

Analysis populations and the handling of missing data were pre‐specified and are applied consistently throughout this article. Of the 120 enrolled participants, 115 attended all five visits and contributed complete data; five did not complete follow‐up. Of these five, three were lost to follow‐up (two relocated and one had an intercurrent illness unrelated to the study) and two discontinued the intervention before the 12‐week visit because their diary‐recorded adherence fell below the pre‐specified 75% threshold; none of the five attended the 12‐week (T4) visit, so no T4 outcome data were available for any of them. The primary analysis population was therefore the completer sample of 115 participants with complete paired data at every visit. All paired *t*‐tests and Wilcoxon tests, all correlation analyses, the multiple linear regression and the repeated‐measures ANOVA (RM‐ANOVA) were performed on these 115 complete cases; consequently, every paired comparison, correlation and the regression is based on *n* = 115 (paired *t* df = 114; regression *F* df as reported in Section 3.7), and each table and figure states *n* = 115. As a sensitivity analysis addressing the five non‐completers, a mixed‐effects model for repeated measures (MMRM) was fitted to all 120 enrolled participants using all available observations under a missing‐at‐random assumption without single‐value imputation. No last‐observation‐carried‐forward imputation was used. Baseline (T0) data were available for all 120 enrolled participants; the number of observed measurements available at each visit is reported in Section 3.1 and Table 1. The pre‐specified analysis plan is as follows:

**Table 1:** Baseline characteristics and available measurements (primary analysis population, *n* = 115 unless otherwise stated).

Characteristic	Value
Enrolled, *n*	120
Completed all five visits (primary analysis population), *n*	115
Age, years, mean (SD) (range)	26.4 (4.7) (18–35)
Sex, female, *n* (%)	61 (53.0%)
Sex, male, *n* (%)	54 (47.0%)
Teeth present, mean (SD) (range)	27.8 (1.4) (24–32)
Brushing twice daily, *n* (%)	76 (66.1%)
Interdental cleaning, regular, *n* (%)	17 (14.8%)
Baseline MGI, mean (SD) (range)	1.78 (0.41) (1.02–2.48)
Baseline plaque index, mean (SD)	1.88 (0.49)
Baseline bleeding on probing, mean (SD), % sites	27.9 (8.7)
Baseline organoleptic score, mean (SD) (0–5)	2.21 (0.79)
Baseline BRSP score, mean (SD) (0–10)	5.48 (1.84)
Baseline WHO‐adapted composite, mean (SD)	23.6 (5.3)
Adherence, mean (SD), % prescribed doses	92.7 (7.1)
Observed measurements available, T0/T1/T2/T3/T4	120/120/118/117/115

*Note:* WHO‐adapted composite: a higher score denotes better oral hygiene and self‐perceived gingival health. Percentages for sex are of the *n* = 115 completer population. The final row reports the number of observed measurements available at each visit across the 120 enrolled participants. BRSP, breath‐related self‐perception scale (adapted); MGI, modified gingival index (Lobene et al. [21]).

Abbreviation: SD, standard deviation.

• Pre‐ and post‐comparisons (baseline versus 12 weeks) used paired *t*‐tests for normally distributed outcomes and Wilcoxon signed‐rank tests otherwise, on the 115 completers. Standardised effect sizes (Cohen’s dz, benchmarks 0.20, 0.50 and 0.80 for small, medium and large) and 95% confidence intervals for the within‐subject mean difference were reported.
• Time‐course comparisons across the five timepoints used one‐way RM‐ANOVA on the 115 completers, with Mauchly’s test for sphericity and the Greenhouse and Geisser correction where sphericity was violated. Partial eta‐squared was reported; adjacent‐timepoint pairwise comparisons used Bonferroni‐adjusted contrasts.
• To provide an interpretable, sign‐consistent analysis, all change scores were expressed as improvement magnitudes: for MGI, PI, BOP, the organoleptic score and the BRSP scale, improvement was defined as baseline minus 12 weeks (T0 minus T4); for the WHO‐adapted composite, where a higher score is better, improvement was defined as 12 weeks minus baseline (T4 minus T0). Under this convention, a positive value denotes improvement for every outcome, and positive correlations and regression coefficients denote concordant improvement.
• Associations between improvement magnitudes in clinical indices and patient‐reported outcomes used Pearson correlation coefficients for normally distributed differences and Spearman coefficients otherwise (*n* = 115).
• Multiple linear regression evaluated independent predictors of the primary improvement magnitude (MGI improvement, T0 minus T4), with pre‐specified covariates: age, sex, baseline MGI, baseline WHO‐adapted composite and adherence proportion. Standardised regression coefficients (beta) are reported with 95% confidence intervals. The confidence interval for each standardised coefficient was computed as the coefficient plus or minus the product of its standard error and the critical *t* value at the residual degrees of freedom (df = 109; two‐sided *t* of 1.982), using the standard errors obtained after refitting the model on *z*‐standardised predictor and outcome variables, so that the reported intervals correspond directly to the standardised coefficients.

All hypothesis tests were two‐sided; a *p* below 0.05 was considered statistically significant. Because all comparisons are within‐subject and uncontrolled, *p*‐values and effect sizes are interpreted descriptively, as indicators of the magnitude and consistency of within‐subject change, and not as evidence of a controlled treatment effect.

## 3. Results

### 3.1. Participant Flow, Analysis Populations and Baseline Characteristics

Of 154 adults screened for eligibility during the recruitment window (7 July to 22 August 2025), 34 were excluded (21 did not meet inclusion criteria, 9 declined to participate and 4 for other reasons) and 120 participants were enrolled and received the intervention. Complete outcome data at all five visits were obtained for 115 participants (95.8%), who constituted the primary (completer) analysis population. Of the five who did not complete, three were lost to follow‐up (two relocated and one intercurrent illness unrelated to the study) and two discontinued before the 12‐week visit because diary‐recorded adherence fell below the pre‐specified 75% threshold; none of the five attended the T4 visit, so none contributed a 12‐week outcome. The number of observed measurements available was 120 at T0, 120 at T1, 118 at T2, 117 at T3 and 115 at T4 (Table 1). Baseline characteristics of the primary analysis population are summarised in Table 1.

Adherence in the completer population was high on the basis of diary records: mean (SD) self‐reported intake was 92.7% (7.1) of prescribed doses. As stated in the Section 2, adherence was self‐reported and was not biologically verified.

### 3.2. Distributional Checks and Choice of Test

Shapiro and Wilk tests on the within‐subject T0‐to‐T4 difference scores in the completer sample were consistent with normality for MGI (*W* = 0.987, *p* = 0.33), PI (*W* = 0.991, *p* = 0.48), BOP (*W* = 0.984, *p* = 0.22) and the WHO‐adapted composite (*W* = 0.990, *p* = 0.46), justifying paired *t*‐tests. The organoleptic score (*W* = 0.969, *p* = 0.04) and BRSP (*W* = 0.972, *p* = 0.05) showed mild departures from normality; results for these outcomes are reported from both paired *t*‐tests and Wilcoxon signed‐rank tests, which were concordant. Mauchly’s test was significant for MGI, PI and BOP, and the Greenhouse and Geisser correction was applied for the repeated‐measures analyses accordingly.

### 3.3. Primary Outcome: Gingival Inflammation

In the primary completer analysis (*n* = 115), mean MGI decreased from 1.78 (SD 0.41) at baseline to 0.96 (SD 0.36) at 12 weeks. The within‐subject reduction of 0.82 units (95% CI: 0.73–0.91; *t* (114) = 18.9, *p* < 0.001) corresponded to a 46% relative decrease and a large standardised within‐subject effect size (Cohen’s dz ~1.76). RM‐ANOVA indicated a significant effect of time across the five visits (*F* (2.9, 330.6) = 180.9, *p* < 0.001, partial eta‐squared = 0.61), with significant Bonferroni‐adjusted reductions between every pair of adjacent visits (all *p* < 0.001). The mixed‐effects sensitivity analysis on all 120 enrolled participants gave a concordant estimated 12‐week reduction of 0.81 units (95% CI: 0.73–0.89), indicating that the five non‐completers did not materially affect the estimate. Because there was no concurrent control group, this trajectory should not be read as evidence that the product alone produced the change; a comparably timed improvement could also reflect the standardised oral hygiene instruction given to all participants, repeated study contact, increased motivation, or regression to the mean.

### 3.4. Secondary Outcomes

All 5 secondary outcomes improved across 12 weeks in the completer analysis (Table 2). PI decreased by 0.81 units (43%; *t* (114) = 17.7, *p* < 0.001; dz ~1.65). BOP decreased from 27.9% to 13.1% of sites, an absolute reduction of 14.8 percentage points (53% relative; *t* (114) = 15.3, *p* < 0.001; dz ~1.43). The organoleptic score declined by 1.03 units (47%; paired *t* (114) = 14.6, *p* < 0.001; Wilcoxon *Z* = 9.10, *p* < 0.001; dz ~1.36), and the BRSP score improved (decreased) by 2.34 units (43%; paired *t* (114) = 13.6, *p* < 0.001; Wilcoxon *Z* = 8.96, *p* < 0.001; dz ~1.27). The WHO‐adapted composite increased by 7.3 points (+ 31%; *t* (114) = 16.1, *p* < 0.001; dz ~1.50). These are within‐subject changes in an uncontrolled study and are interpreted descriptively.

**Table 2:** Pre and post within‐subject change at baseline and 12 weeks (primary completer analysis, *n* = 115).

Outcome	Baseline, mean (SD)	12 weeks, mean (SD)	Mean Δ	95% CI for Δ	Relative change (%)	*t* (114)	Cohen’s dz
Modified gingival index (MGI)	1.78 (0.41)	0.96 (0.36)	0.82	0.73–0.91	46	18.9	1.76
Plaque index (Silness and Löe)	1.88 (0.49)	1.07 (0.44)	0.81	0.72–0.90	43	17.7	1.65
Bleeding on probing (% sites)	27.9 (8.7)	13.1 (7.2)	14.8	12.9–16.7	53	15.3	1.43
Organoleptic score (0– 5)	2.21 (0.79)	1.18 (0.62)	1.03	0.89–1.17	47	14.6	1.36
BRSP score (0–10)	5.48 (1.84)	3.14 (1.51)	2.34	2.00–2.68	43	13.6	1.27
WHO‐adapted composite	23.6 (5.3)	30.9 (4.8)	+ 7.30	6.40–8.20	+ 31	16.1	1.50

*Note:* Paired *t*‐test (two‐sided), *n* = 115, df = 114. Δ = mean within‐subject change; 95% CI = confidence interval for the mean difference. Cohen’s dz = mean difference divided by the SD of the difference scores; benchmarks 0.20, 0.50 and 0.80. A reduction indicates improvement for all outcomes except the WHO‐adapted composite, where a higher score is better (shown as +). Effect sizes and *p*‐values describe within‐subject change only and do not reflect a controlled treatment effect. All values are observed (not model‐derived); the mixed‐effects sensitivity estimate for MGI is given in Section 3.3.

### 3.5. Time‐Course of Change Across Five Visits

RM‐ANOVA on the completer sample showed a significant effect of time for all six outcomes (all *p* < 0.001; Table 3), with partial eta‐squared from 0.46 to 0.61. Measurable within‐subject differences were present by the 1‐week visit and continued through 12 weeks. An early or progressive within‐subject trajectory is not interpreted as demonstrating biological activity of the product because the same pattern is compatible with reinforcement of oral hygiene behaviour under standardised instruction, repeated study contact and regression to the mean in the absence of a control group.

**Table 3:** Repeated‐measures ANOVA: means (SD) across five visits and omnibus statistics (completer analysis, *n* = 115).

Outcome	T0	T1	T2	T3	T4	Mauchly *W* (*p*)	*F* (GG df)	*η* ^2^ *p*
MGI	1.78 (0.41)	1.54 (0.39)	1.28 (0.37)	1.10 (0.36)	0.96 (0.36)	0.71 (<0.001)	180.9 (2.9, 330.6)	0.61
Plaque index	1.88 (0.49)	1.63 (0.47)	1.35 (0.46)	1.18 (0.45)	1.07 (0.44)	0.88 (<0.001)	166.7 (3.0, 342.0)	0.59
Bleeding on probing (% sites)	27.9 (8.7)	23.6 (8.3)	19.2 (7.9)	15.8 (7.6)	13.1 (7.2)	0.76 (<0.001)	140.2 (2.8, 319.2)	0.55
Organoleptic score	2.21 (0.79)	1.95 (0.76)	1.62 (0.72)	1.36 (0.67)	1.18 (0.62)	0.92 (0.004)	95.4 (3.1, 353.4)	0.46
BRSP	5.48 (1.84)	4.89 (1.76)	4.21 (1.68)	3.63 (1.59)	3.14 (1.51)	0.90 (0.002)	102.8 (2.7, 307.8)	0.47
WHO‐adapted composite	23.6 (5.3)	25.9 (5.1)	27.9 (4.9)	29.4 (4.8)	30.9 (4.8)	0.93 (0.008)	156.4 (3.0, 342.0)	0.58

*Note:* Values are observed means (SD). GG = Greenhouse and Geisser correction applied where Mauchly’s test was significant; corrected df shown. Bonferroni‐adjusted contrasts between all adjacent visit pairs were significant at *p* < 0.001 for all outcomes. The final MGI SD (0.36) is consistent with Table 2. *η* ^2^ *p*  =  partial eta‐squared; benchmarks 0.01, 0.06 and 0.14.

### 3.6. Correlations Between Improvement Magnitudes

To keep a single sign convention, all change scores were expressed as improvement magnitudes (Section 2.10), so that a positive coefficient denotes concordant improvement for every pair. Improvement in MGI correlated moderately and positively with improvement in PI (*r* = 0.62, 95% CI: 0.50–0.72, *p* < 0.001) and in BOP (*r* = 0.58, 95% CI: 0.45–0.69, *p* < 0.001), and with improvement in the WHO‐adapted composite (Spearman rho = 0.41, *p* < 0.001), so that participants whose oral hygiene knowledge and practices improved most also showed the largest reductions in gingival inflammation. Improvement in self‐perceived breath (BRSP) correlated with improvement in the organoleptic score (rho = 0.55, *p* < 0.001). The full matrix is shown in Table 4; all off‐diagonal coefficients are positive under the improvement‐magnitude convention.

**Table 4:** Correlations among improvement magnitudes (completer analysis, *n* = 115).

Improvement	MGI	PI	BOP	Organoleptic	BRSP	WHO
MGI	1.00	0.62 ^∗∗∗^	0.58 ^∗∗∗^	0.49 ^∗∗∗^	0.42 ^∗∗∗^	0.41 ^∗∗∗^
PI	0.62 ^∗∗∗^	1.00	0.51 ^∗∗∗^	0.43 ^∗∗∗^	0.39 ^∗∗∗^	0.36 ^∗∗∗^
BOP	0.58 ^∗∗∗^	0.51 ^∗∗∗^	1.00	0.40 ^∗∗∗^	0.34 ^∗∗∗^	0.30 ^∗∗∗^
Organoleptic	0.49 ^∗∗∗^	0.43 ^∗∗∗^	0.40 ^∗∗∗^	1.00	0.55 ^∗∗∗^	0.27 ^∗∗^
BRSP	0.42 ^∗∗∗^	0.39 ^∗∗∗^	0.34 ^∗∗∗^	0.55 ^∗∗∗^	1.00	0.25 ^∗∗^
WHO	0.41 ^∗∗∗^	0.36 ^∗∗∗^	0.30 ^∗∗∗^	0.27 ^∗∗^	0.25 ^∗∗^	1.00

*Note:* Pearson *r* for normally distributed pairs, Spearman rho otherwise. All variables are improvement magnitudes (Section 2.10): for MGI, PI, BOP, Organoleptic and BRSP, improvement = T0 minus T4; for the WHO‐adapted composite, improvement = T4 minus T0. Under this convention every off‐diagonal coefficient is positive, denoting concordant improvement. Diagonal entries are 1.00.

^∗∗^ *p* < 0.01.

^∗∗∗^ *p* < 0.001.

### 3.7. Predictors of Improvement in Gingival Inflammation

A multiple linear regression identified independent predictors of the primary improvement magnitude (MGI improvement, defined as T0 minus T4, so that a larger positive value denotes greater improvement), with covariates age, sex, baseline MGI, baseline WHO‐adapted composite and adherence. The model was significant (*F* (5, 109) = 27.4, *p* < 0.001; *R*‐squared = 0.557, adjusted R‐squared = 0.537). All coefficients below are standardised regression coefficients (beta), obtained by fitting the model to *z*‐standardised predictor and outcome variables; each 95% confidence interval was computed as the standardised coefficient plus or minus its standard error multiplied by the critical *t* value at the residual degrees of freedom (df = 109). Higher baseline MGI independently predicted greater improvement (standardised beta = + 0.41, 95% CI: + 0.31 to + 0.51, *p* < 0.001), consistent both with greater scope for improvement in more inflamed participants and with regression to the mean. Higher adherence independently predicted greater improvement (standardised beta = + 0.27, 95% CI: + 0.16 to + 0.38, *p* < 0.001), and a higher baseline behavioural composite predicted marginally greater improvement (standardised beta = + 0.14, 95% CI: + 0.04 to + 0.24, *p* = 0.008). Age (standardised beta = + 0.04, 95% CI: −0.07 to + 0.15, *p* = 0.46) and sex (standardised beta = + 0.03, 95% CI: −0.09 to + 0.15, *p* = 0.61) were not independent predictors. Variance inflation factors were all below 1.6, indicating no problematic multicollinearity.

### 3.8. Primary and Sensitivity Analyses

The primary analysis was the completer analysis (*n* = 115) reported above. One sensitivity analysis was pre‐specified to address the five non‐completers: a MMRM on all 120 enrolled participants, modelling visit as a categorical fixed effect and participant as a random intercept under a missing‐at‐random assumption. This model returned an estimated 12‐week MGI of 0.97 (95% CI: 0.90–1.04) and an estimated overall reduction of 0.81 units (95% CI: 0.73–0.89), concordant with the primary completer estimate of 0.82 units. Because the mixed‐effects model uses each participant’s available longitudinal records rather than complete pairs, it does not create paired observations for the completer‐based paired tests, correlations or change‐score regression; those analyses are reported only on the 115 complete cases, and the mixed‐effects model is presented solely as a sensitivity check on the primary MGI estimate. The concordance between the two indicates that the small amount of missing data did not drive the result.

### 3.9. Safety and Tolerability

No product‐related adverse events were recorded across the 12‐week follow‐up. Seven adverse events of any kind were reported, occurring in seven different participants (5.8% of the enrolled sample): three episodes of transient nausea, two of mild headache, one common cold and one episode of gastrointestinal upset. Each was mild and self‐limiting, none led to withdrawal and none was assessed as related to the investigational product. The nausea and gastrointestinal events were judged unrelated on the basis of standard criteria: each began more than a week after continuous lozenge use without any change in the regimen, each had an identifiable alternative explanation (dietary indiscretion in two cases and a self‐limiting viral gastroenteritis reported by a household contact in one), each resolved without interruption of lozenge use (so that continued use did not reproduce the symptom), and none was accompanied by any local oral or mucosal reaction at the site of application. The five participants who did not complete follow‐up did so for reasons unrelated to the product (two relocated, one intercurrent illness unrelated to the study, and two discontinued the intervention because diary‐recorded adherence fell below the pre‐specified 75% threshold). The two low‐adherence participants discontinued the lozenge and did not attend the 12‐week visit; they therefore had no T4 outcome data and were not part of the completer analysis rather than being excluded despite having complete data. The lozenge was reported by participants to be palatable and easy to use; this was not formally quantified and is reported descriptively.

## 4. Discussion

In this 12‐week, single‐arm, pre‐ and post‐interventional study, twice‐daily use of a polyherbal slow‐dissolving buccal lozenge (CBFP‐ODS) was associated with within‐subject improvements across all measured domains of gingival health in young adults with mild‐to‐moderate gingivitis. Objective clinical indices (MGI, PI and BOP), an examiner‐rated measure of oral malodour, a patient‐reported breath measure and a behavioural composite all moved coherently in the expected direction across five visits, with moderate cross‐outcome correlations. Because the study had no control group, this internal coherence indicates a consistent within‐subject pattern rather than a demonstrated biological or behavioural effect of the product; the observed change cannot be separated from improved oral hygiene behaviour under standardised instruction, participation and expectancy effects, regression to the mean or natural clinical variation.

### 4.1. Findings in Context

The within‐subject MGI change observed here (0.82 units; 46% over 12 weeks) is at the upper end of the range reported in published curcumin‐based gingival studies, in which gingival‐index reductions of roughly 0.3–0.6 units over shorter 2–6 week windows have generally been reported [11, 12]. Two features may be relevant to the larger observed change: a longer 12‐week window with an everyday‐use format and a delivery vehicle intended to prolong contact with the gingival margin. These are presented as possible contributing factors rather than established explanations because in an uncontrolled study, a general improvement in oral hygiene over the period is an equally plausible contributor. Any reference to enhanced local exposure remains conditional and hypothetical, as no absorption, tissue‐concentration or pharmacokinetic endpoints were measured; the buccal format is consistent with pharmaceutical evidence that prolonged‐contact delivery can improve local retention of mucosally active agents relative to rapidly cleared formats [13], but this study does not itself demonstrate such an effect.

The plaque change observed (0.81 units in PI; 43%) lies within the ranges reported for various plaque‐control regimens. We deliberately avoid comparing this within‐subject change against the magnitude of plaque reduction reported for chlorhexidine because no chlorhexidine arm was included and any such comparison could wrongly imply comparable efficacy. The conceptual point is that chlorhexidine, although effective, is limited for sustained daily preventive use by staining, taste alteration and mucosal effects [5, 7], which is why a daily‐use botanical format aimed at a different everyday niche merits controlled evaluation.

### 4.2. Biological Plausibility

The clinical observations are compatible with mechanisms reported for the constituent botanicals (Figure 2), which are presented as biological plausibility to be tested and not as mechanisms demonstrated here. Curcumin has been reported to inhibit NF‐κB signalling, COX‐2 expression and pro‐inflammatory cytokines including IL‐1β and TNF‐α [8–10], and its reported effects on oxidative stress are consistent with the direction of change in BOP [10]. The other botanicals contribute complementary reported actions: *Ocimum sanctum* antimicrobial and immunomodulatory activity [16], *Zingiber officinale* antioxidant and prostaglandin‐modulating effects [17], *Cinnamomum verum* anti‐adhesive and antimicrobial properties [18], *Mentha piperita* mucosal comfort and antimicrobial activity, and *Piper longum* the bioavailability‐enhancing action of piperine [14, 15]. Whether these mechanisms mediated the changes observed here cannot be determined from clinical indices alone and would require dedicated biomarker, microbiological and pharmacokinetic endpoints (Figure 3).

**Figure 2:**
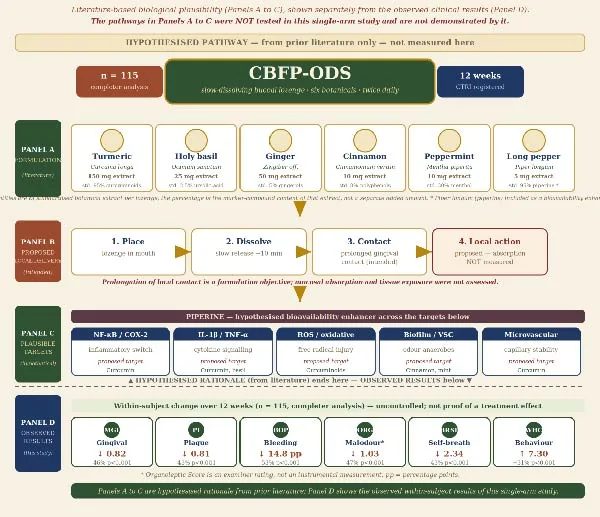
Proposed (hypothetical) mechanistic rationale for CBFP‐ODS. Panels A to C summarise literature‐based biological plausibility (the botanical formulation, the intended local‐delivery sequence and proposed molecular and cellular targets) and are explicitly labelled as hypothesised and not measured in this study. A dashed separator divides this rationale from Panel D, which shows the observed within‐subject results of the present single‐arm study (*n* = 115). The proposed pathways were not tested here; prolongation of local contact is a formulation objective, and mucosal absorption and tissue exposure were not assessed. The organoleptic score is an examiner rating, not an instrumental measurement. pp, percentage points.

**Figure 3:**
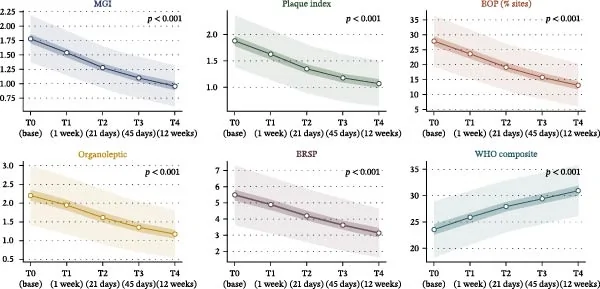
Observed temporal trajectories across 12 weeks (completer analysis, *n* = 115). Points are observed means; dark bands show the 95% confidence interval and light bands 1 SD. The plotted values are observed (not model‐derived). Repeated‐measures ANOVA was significant for all six outcomes (*p* < 0.001). Trajectories describe within‐subject change in an uncontrolled design and are not evidence of a controlled treatment effect. BRSP, breath‐related self‐perception; MGI, modified gingival index. A higher WHO‐adapted composite denotes better oral hygiene and self‐perceived gingival health.

### 4.3. Halitosis and Patient‐Reported Outcomes

Improvement in the examiner‐rated organoleptic score alongside the BRSP scale is a pattern under‐reported in gingivitis studies. Oral malodour is linked to gingival inflammation and to volatile sulphur compound‐producing anaerobes, so concurrent change in malodour alongside plaque and gingival inflammation is internally coherent and aligns with prior work [23]. The moderate correlation between the examiner‐rated and self‐perceived breath measures (rho = 0.55 between improvement scores) is described here as an exploratory association only; it does not establish the concurrent validity of the adapted questionnaire, which would require formal validation against an instrumental standard. The organoleptic score is an examiner rating rather than an instrumental measurement and is described consistently as such throughout. The simultaneous rise in the WHO‐adapted behavioural composite is consistent with a daily‐use format acting as a behavioural cue [24], although this cannot be separated from the standardised instruction given to all participants [25, 26].

### 4.4. Wellness Positioning and Translational Significance

Gingivitis remains among the most prevalent oral conditions globally and is, in many populations, under‐detected and under‐managed [1, 2, 4]. Within the wider movement toward preventive, non‐invasive oral health strategies, there may be room for a daily‐use functional oral care category between mechanical plaque control and short‐course chemical adjuncts. The present findings are consistent with the potential of polyherbal slow‐dissolving lozenges within such a category but do not establish it. Any statement about a daily‐use clinical role, and about the safety of sustained use, remains explicitly conditional: this uncontrolled study cannot establish a comparative safety advantage or the suitability of long‐term use, and the intervention is positioned only as a candidate wellness adjunct for use alongside, and not instead of, established oral hygiene practice and professional dental care, pending controlled trials [27, 28].

### 4.5. Strengths

Strengths include an adequately powered sample for its primary within‐subject contrast, a five‐timepoint repeated‐measures design, the use of established clinical indices alongside patient‐reported measures, single‐examiner calibration with reported reliability coefficients, prospective registration and structured reporting [19], and a pre‐specified analysis plan with a clearly identified primary analysis, a concordant sensitivity analysis, effect sizes and confidence intervals, and a covariate‐adjusted regression.

### 4.6. Limitations

Several limitations materially constrain the interpretation. First, the study was uncontrolled, non‐randomised and open‐label, with no placebo lozenge, no active comparator and no participant or examiner blinding; the single‐arm design precludes causal inference, and the within‐subject change cannot be partitioned between any biological action of the intervention, the standardised oral hygiene instruction given to all participants, the behavioural reinforcement of participation (Hawthorne effect), regression to the mean and natural variation. Second, no biomarker, salivary, microbiological, absorption or volatile sulphur compound endpoints were measured, so no mechanistic or bioavailability claim can be supported. Third, oral malodour was assessed by examiner rating only [29]. Fourth, two patient‐reported instruments were adapted rather than previously validated. Fifth, adherence was self‐reported and not biologically verified. Sixth, all assessments were by a single examiner, precluding inter‐examiner reliability checks and carrying a residual single‐rater risk. Seventh, the population was restricted to adults aged 18–35 from a single urban catchment, limiting generalisability to older adults, to people with severe gingivitis or periodontitis, and to medically complex populations. Finally, this study cannot establish any comparative tolerability or safety advantage over chlorhexidine or the suitability of sustained daily use.

### 4.7. Implications for Further Research

These findings support the case for, but do not establish, the efficacy of CBFP‐ODS. The necessary next step is a randomised, controlled, blinded trial comparing CBFP‐ODS against a matched placebo lozenge, ideally with a short‐term chlorhexidine reference arm and blinded outcome assessment. Future trials should incorporate microbiological and salivary biomarker assays and instrumental volatile sulphur compound measurement, formally validate the patient‐reported instruments used here, and extend follow‐up to assess durability and relapse. Evaluation in specific populations, including orthodontic patients, individuals with peri‐implant mucositis and people with limited access to professional dental care, would help define where in the preventive‐care pathway this format might fit.

## 5. Conclusion

In adults aged 18–35 years with mild‐to‐moderate gingivitis, twice‐daily use of CBFP‐ODS, a sugar‐free slow‐dissolving buccal lozenge combining six standardised botanicals, was associated with within‐subject improvements in gingival inflammation, plaque accumulation, gingival bleeding, oral malodour and oral hygiene behaviour over 12 weeks. Because the study was single‐arm, uncontrolled and open‐label, these observations reflect within‐subject improvement and do not demonstrate efficacy; they do not verify transmucosal absorption, and they cannot separate the intervention from improved oral hygiene behaviour, participation effects, regression to the mean or natural variation. Randomised, placebo‐controlled and active‐controlled trials with blinded assessment and mechanistic endpoints are required to determine whether CBFP‐ODS has a genuine therapeutic effect.

## Funding

The authors received no specific funding from any public, commercial or not‐for‐profit body. The costs of preparing and supplying the trial lozenges were met by the investigators.

## Disclosure

TREND statement for non‐randomised interventional studies, supported by CONSORT 2010 items applicable to a single‐arm design. This was an investigator‐initiated study.

## Ethics Statement

This study was conducted in accordance with the Declaration of Helsinki (2013 revision). The protocol was approved by the Institutional Ethics Committee of Dr. G. D. Pol Foundation Y.M.T. Dental College and Hospital, Kharghar, Navi Mumbai (Indian Council of Medical Research and Department of Health Research registration EC/NEW/INST/2023/3747), under Reference YMTDC/IEC/OUT/2025/329‐P, dated 12 June 2025. The trial was prospectively registered with the Clinical Trials Registry, India. Written informed consent was obtained from all participants prior to any study procedure.

## Conflicts of Interest

The authors declare no conflicts of interest.

## Supporting Information

Additional supporting information can be found online in the Supporting Information section.

## Supporting information


**Supporting Information** Table S1: Composition, standardisation and batch quality control of the investigational product, including the per‐lozenge quantity and marker‐compound standardisation of each botanical extract, the excipient base, the identity and assay methods (thin‐layer chromatography and high‐performance liquid chromatography), the manufacturer and manufacturing‐licence details, and the batch acceptance criteria. Cited in Section 2.5.1. Appendix S1: The complete breath‐related self‐perception (BRSP) scale: all six items, the 0–10 response anchors, the reverse‐scored items and the averaging rule used to derive the total score. Cited in Section 2.6.2. Appendix S2: The complete WHO‐adapted Gingival Health and Oral Hygiene Questionnaire (composite): all twelve items across the knowledge, practice and self‐perception domains, the 0–3 response coding, the summation and the rescaling to the 0–40 reporting range. Cited in Section 2.6.2. Table S2: The a priori sample‐size calculation (G ^∗^Power Version 3.1), reporting the test family, effect size, alpha, target power, noncentrality parameter, critical *t*, degrees of freedom, required completer sample and achieved power. Cited in Section 2.9.

## Data Availability

The de‐identified dataset supporting the findings of this study is available from the corresponding author upon reasonable request.
