# Supplementary material for: A Curcumin‐Based Polyherbal Slow‐Dissolving Buccal Lozenge (CBFP‐ODS) and Changes in Gingival Inflammation, Plaque Accumulation, Bleeding on Probing and Oral Malodour: A 12‐Week Prospective Single‐Arm Interventional Study in Young Adults
Source: Int J Dent. 2026 Nov 10;2026:2236774. doi: 10.1155/ijod/2236774 (PMC13749427; doi:10.1155/ijod/2236774)
Supplement: Supplementary file 1 [file IJOD-2026-2236774-s001.docx]

**SUPPLEMENTARY MATERIAL**

*A Curcumin-Based Polyherbal Slow-Dissolving Buccal Lozenge (CBFP-ODS) and Changes in Gingival Inflammation, Plaque Accumulation, Bleeding on Probing, and Oral Malodour*

Trial registration CTRI/2025/06/088964.

This Supplementary Material provides the batch quality-control and manufacturing specifications of the investigational product (Supplementary Table S1), the complete patient-reported instruments with their scoring rules (Supplementary Appendix S1 and S2), and the a priori sample-size calculation output (Supplementary Table S2).

# **Supplementary Table S1. Composition, standardisation and batch quality control of the investigational product**

| **Item** | **Specification** |
| --- | --- |
| **Product name** | Dennkur (CBFP-ODS research formulation) |
| **Dosage form** | Sugar-free, slow-dissolving buccal lozenge |
| **Loan licensee** | Chokhani Pharma, Unit B of 306, Kakad Chambers, 132, Dr. A. B. Road, Worli, Mumbai 400018, Maharashtra, India |
| **Manufactured at** | Survey No. 338 (P-38), Kalmeshwar, Nagpur 441501, Maharashtra, India (c/o Unijules Life Sciences Ltd) |
| **Manufacturing licence** | MH/104676A (Form 25-E) |
| **Product permission** | 1098840, dated 09 August 2022 |
| **Pharmacopoeial reference** | Ayurvedic Pharmacopoeia of India |
| **Curcuma longa extract** | 150 mg per lozenge; not less than 95% total curcuminoids (approximately 142.5 mg curcuminoids) |
| **Ocimum sanctum extract** | 25 mg per lozenge; not less than 2.5% ursolic acid |
| **Zingiber officinale extract** | 50 mg per lozenge; not less than 5% gingerols |
| **Cinnamomum verum extract** | 10 mg per lozenge; not less than 8% total polyphenols |
| **Mentha piperita extract** | 10 mg per lozenge; not less than 30% total menthol |
| **Piper longum extract** | 5 mg per lozenge; not less than 95% piperine (approximately 4.75 mg piperine) |
| **Total botanical extract load** | Approximately 250 mg per lozenge |
| **Excipient base** | Isomalt and sorbitol (bulk sweeteners); acacia and hydroxypropyl methylcellulose (matrix formers); magnesium stearate (lubricant); natural peppermint flavour |
| **Identity testing** | Thin-layer chromatography for each botanical extract |
| **Assay (marker compounds)** | High-performance liquid chromatography (HPLC) for total curcuminoids and piperine, per batch, against reference standards |
| **Uniformity testing** | Weight, hardness, appearance and disintegration time checked per batch |
| **Acceptance (weight)** | Mean lozenge weight within plus or minus 5% of the target weight |
| **Acceptance (curcuminoids)** | Not less than 95% of the labelled curcuminoid content per batch |
| **Acceptance (piperine)** | Not less than 90% of the labelled piperine content per batch |
| **Acceptance (disintegration)** | Slow dissolution over approximately 8 to 12 minutes in the buccal cavity |

Quantities are of standardised botanical extract per lozenge; the stated percentage is the marker-compound content of that extract (verified per batch), not a separately added quantity of the pure marker compound. A commercial oral-care product later derived from this delivery platform uses a reformulated botanical composition and is not identical to the trial formulation described here.

# **Supplementary Appendix S1. Breath-Related Self-Perception (BRSP) scale**

**Format.** Six self-rated items. Each item is scored on an 11-point numerical scale from 0 to 10. Items 1 and 5 are worded positively and are reverse-scored (10 minus the response) before combination, so that a higher final score consistently denotes worse self-perceived breath. The six item scores (after reverse-scoring where indicated) are averaged to give a total scale score from 0 to 10.

**Anchors.** For each item, 0 = strongly disagree and 10 = strongly agree.

**Items**

1. I feel confident that my breath is fresh during close conversation.

2. I worry that other people notice an odour from my mouth.

3. I avoid speaking closely to others because of concern about my breath.

4. I feel self-conscious about my breath in social or work settings.

5. I check my breath (for example, by cupping my hand) during the day.

6. Overall, I am dissatisfied with the freshness of my breath.

**Scoring.** Reverse-score items 1 and 5 (10 minus response). Total BRSP score = mean of the six item scores (range 0 to 10). Higher = worse self-perceived breath. Internal consistency at baseline (T0): Cronbach's alpha 0.82.

# **Supplementary Appendix S2. WHO-adapted Gingival Health and Oral Hygiene Questionnaire (composite)**

**Format.** Twelve items across three domains (knowledge, practice and self-perception), adapted from the oral health questionnaire structure of the WHO Oral Health Surveys (Basic Methods, 5th edition). Knowledge items (1 to 4) are scored 0 (incorrect or do not know) or correct on a graded 0 to 3 scale. Practice items (5 to 8) are scored 0 to 3 by frequency (0 = never, 1 = rarely, 2 = sometimes, 3 = always or daily as appropriate). Self-perception items (9 to 12) are scored 0 to 3 by agreement (0 = strongly disagree to 3 = strongly agree). Item scores are summed to a composite from 0 to 36, linearly rescaled to a 0 to 40 reporting range; a higher total denotes better oral hygiene knowledge, practice and self-perceived gingival health.

**Items**

1. I know that bleeding gums are a sign of gum inflammation. (knowledge)

2. I know that dental plaque is the main cause of gum disease. (knowledge)

3. I know that brushing twice a day helps prevent gum problems. (knowledge)

4. I know that cleaning between the teeth is important for gum health. (knowledge)

5. I brush my teeth twice a day. (practice)

6. I clean between my teeth (interdental brush or floss) daily. (practice)

7. I replace my toothbrush regularly. (practice)

8. I attend a dental check-up when advised. (practice)

9. My gums feel healthy and do not bleed when I clean them. (self-perception)

10. My mouth feels clean and fresh through the day. (self-perception)

11. I am satisfied with the appearance of my gums. (self-perception)

12. I feel my current oral care routine is effective. (self-perception)

**Scoring.** Sum all 12 items (each 0 to 3) for a raw score of 0 to 36, then rescale to 0 to 40 (raw multiplied by 40 divided by 36). Higher = better oral hygiene and self-perceived gingival health. Internal consistency at baseline (T0): Cronbach's alpha 0.79.

# **Supplementary Table S2. A priori sample-size calculation (G*Power version 3.1)**

| **Parameter** | **Value** |
| --- | --- |
| **Test family** | t tests |
| **Statistical test** | Means: difference between two dependent means (matched pairs) |
| **Type of power analysis** | A priori: compute required sample size |
| **Tails** | Two |
| **Effect size dz** | 0.288 |
| **Alpha (two-sided)** | 0.05 |
| **Target power (1 minus beta)** | 0.80 |
| **Noncentrality parameter delta** | 2.836 |
| **Critical t** | 1.985 |
| **Degrees of freedom** | 96 |
| **Required completer sample size** | 97 |
| **Actual power at n = 97** | 0.802 |
| **Enrolled (allowing approximately 20% attrition)** | 120 |
| **Achieved completers** | 115 |
| **Post hoc power at n = 115 (dz = 0.288)** | Approximately 0.86 |

The effect size dz = 0.288 was derived from an assumed within-subject mean change of 0.15 MGI units and a difference-score standard deviation of 0.52 MGI units (0.15 divided by 0.52), taken from the within-group pre-to-post change and dispersion reported for the curcumin arms of prior controlled gingival trials. These were the protocol assumptions specified before recruitment.
